# Nitro-Oxidative Stress after Neuronal Ischemia Induces Protein Nitrotyrosination and Cell Death

**DOI:** 10.1155/2013/826143

**Published:** 2013-08-01

**Authors:** Marta Tajes, Gerard ILL-Raga, Ernest Palomer, Eva Ramos-Fernández, Francesc X. Guix, Mònica Bosch-Morató, Biuse Guivernau, Jordi Jiménez-Conde, Angel Ois, Fernando Pérez-Asensio, Mario Reyes-Navarro, Carolina Caballo, Ana M. Galán, Francesc Alameda, Ginés Escolar, Carlos Opazo, Anna Planas, Jaume Roquer, Miguel A. Valverde, Francisco J. Muñoz

**Affiliations:** ^1^Laboratory of Molecular Physiology and Channelopathies, Universitat Pompeu Fabra, Barcelona, 08003 Catalonia, Spain; ^2^Servei de Neurologia, Hospital del Mar-IMIM-Parc de Salut Mar, 08003 Barcelona, Spain; ^3^Institut d'Investigacions Biomèdiques de Barcelona (IIBB), Consejo Superior de Investigaciones Científicas (CSIC), and Institut d'Investigacions Biomèdiques August Pi i Sunyer (IDIBAPS), 08036 Barcelona, Spain; ^4^Laboratory of Neurobiometals, Department of Physiology, University of Concepción, 5090000 Concepción, Chile; ^5^Hemotherapy and Hemostasis Service, Hospital Clinic, IDIBAPS, University of Barcelona, 08036 Barcelona, Spain; ^6^Servei d'Anatomia Patològica, Hospital del Mar-IMIM-Parc de Salut Mar, 08003 Barcelona, Spain; ^7^Oxidation Biology Laboratory, The Florey Institute of Neuroscience and Mental Health, The University of Melbourne, Parkville, VIC 3010, Australia

## Abstract

Ischemic stroke is an acute vascular event that obstructs blood supply to the brain, producing irreversible damage that affects neurons but also glial and brain vessel cells. Immediately after the stroke, the ischemic tissue produces nitric oxide (NO) to recover blood perfusion but also produces superoxide anion. These compounds interact, producing peroxynitrite, which irreversibly nitrates protein tyrosines. The present study measured NO production in a human neuroblastoma (SH-SY5Y), a murine glial (BV2), a human endothelial cell line (HUVEC), and in primary cultures of human cerebral myocytes (HC-VSMCs) after experimental ischemia *in vitro*. Neuronal, endothelial, and inducible NO synthase (NOS) expression was also studied up to 24 h after ischemia, showing a different time course depending on the NOS type and the cells studied. Finally, we carried out cell viability experiments on SH-SY5Y cells with H_2_O_2_, a prooxidant agent, and with a NO donor to mimic ischemic conditions. We found that both compounds were highly toxic when they interacted, producing peroxynitrite. We obtained similar results when all cells were challenged with peroxynitrite. Our data suggest that peroxynitrite induces cell death and is a very harmful agent in brain ischemia.

## 1. Introduction

 Ischemic stroke, the most common type of stroke, is an acute vascular accident caused by blockage in a brain vessel, which yields to a lack of oxygen supply that dramatically affects the brain parenchyma and brain vasculature. The tissue surrounding the ischemic lesion where neurons are still alive, but their viability that is highly compromised, called the penumbra area, is a major target for ischemic stroke treatments [1, 2].

After ischemia, nitric oxide (NO) is released by endothelial cells to recover blood perfusion [3]. The NO is produced by the NO synthases (NOS), a family of enzymes coded by genes located in different chromosomes [3]. The major regulator of the vascular tone is the endothelium, through the endothelial NOS (eNOS). Diffusion of NO to vascular smooth myocytes produces myorelaxation in a cyclic, guanosine monophosphate-dependent manner [4]. The neuronal (nNOS) and inducible (iNOS) enzymes are expressed in neurons, glial cells, and vascular myocytes [5–12] and may contribute to NO production in ischemic processes. Moreover, a burst of free radicals such as superoxide anion (O_2_^∙−^) [13, 14] is produced after the ischemic event. This scenario could be very harmful because superoxide anion has a high affinity for NO, higher than for the superoxide dismutase [15, 16], and both compounds react with each other and produce peroxynitrite (ONOO^−^) [17]. Peroxynitrite has a gaseous nature that allows it to spread around the surrounding tissue to the ischemic focus. Peroxynitrite provokes protein nitrotyrosination, an irreversible chemical process consisting in the addition of a nitro group (NO_2_) to the tyrosine residues to generate 3-nitrotyrosine [18, 19]. This posttranslational modification is pathological because it usually impairs the physiological function of the proteins [20, 21]. Evidence of protein nitrotyrosination in ischemic stroke has been reported [22].

The present study analyzed the role of the different cell types from brain parenchyma and brain vessels in NO production. Protein expression and mRNA levels of the different NOS were assessed. Moreover, we studied the effects of peroxynitrite on the viability of the different brain cells. 

## 2. Materials and Methods

### 2.1. Cell Cultures

Human neuroblastoma cells (SH-SY5Y) were grown in DMEM (Gibco) supplemented with 15% fetal bovine serum (FBS; Gibco) and antibiotics (100 units/mL penicillin and 10^−6^ *μ*g/mL streptomycin; Gibco). HUVECs were grown in M-199 medium (Gibco) supplemented with 10% FBS, 3.2 mM glutamine (Sigma), and antibiotics. Murine microglial cells (BV2) were grown in RPMI (Gibco) supplemented with 10% FBS and antibiotics. Primary cultures of HC-VSMCs were produced from cerebral arteries (basilar) obtained from autopsies of 4 individuals (55.3 ± 5.6 years) and utilized up to the ten passages [23]. Procedure was approved by the ethics committee of the Institut Mar d'Investigació Mèdica and the Universitat Pompeu Fabra (IMIM-IMAS-UPF). Briefly, pieces of tunica media were incubated with 0.1% collagenase type IV (Sigma) for 35 min at 37°C and cultured to allow HC-VSMC migration to the flask surface. Cells were grown in DMEM with 4.5 g/L glucose (Sigma), 25 mM HEPES (Gibco), 10% FBS, 2.5 *μ*g/mL amphotericin B (Gibco), 100 units/mL penicillin, and 10^−6^ *μ*g/mL streptomycin. Myocytes were characterized by immunostaining with mouse anti-smooth muscle *α*-actin antibody (Ab; Sigma). Cells were used up to ten passages.

### 2.2. Mouse Embryo Hippocampal Cell Cultures

Hippocampal cells were isolated from 18-day-old CB1 mouse embryos. Procedure was approved by the ethics committee of the IMIM-IMAS-UPF. Hippocampi were aseptically dissected and trypsinized. Cells were seeded in DMEM plus 10% horse serum (Gibco) into 1% poly-L-lysine (Sigma) coated coverslips (5 × 10^4^ cells/cover). After 2 h, medium was removed, and Neurobasal medium containing 1% B27 supplement (Gibco) plus antibiotics was added. Glial proliferation was avoided by adding 2 *μ*M cytosine arabinoside (Sigma) at day 3 for 24 h. Cultured hippocampal cells were used for the experiments on day 7, when they were considered to be mature neurons [24].

### 2.3. Human Brain Study

The human cortex sample was obtained from an autopsy of a patient who had a cortical ischemic stroke (Servei d'Anatomia Patològica, Hospital del Mar, Barcelona). The sample was fixed in formalin and embedded in paraffin. Procedure was approved by the ethics committee of the Institut Municipal d'Investigació Mèdica and the Universitat Pompeu Fabra (IMIM-IMAS-UPF).

### 2.4. *In Vitro* Ischemia

Ischemia was induced by an oxygen-glucose deprivation protocol (OGD). Briefly, the culture medium was replaced with a glucose-free balanced saline solution containing 116 mM NaCl, 5.4 mM KCl, 0.8 mM MgSO_4_, 1 mM NaH_2_PO_4_, 26.2 mM NaHCO_3_, and 1.8 CaCl_2_, incubated for 1 h in a nitrogen chamber at 37°C. The OGD medium was then replaced by culture medium, and cells were incubated for up to 12 h or 24 h after OGD under standard culture conditions. 

### 2.5. Free Radical Production

SH-SY5Y cells were seeded on 1.5% gelatin-coated 35 mm coverslips (1 × 10^5^ cells/well) for image analysis with a Leica TCS SP confocal microscope. SH-SY5Y cells were seeded in a 96-well plate (1 × 10^3^ cells/well) for fluorescence quantification with a Fluostar Optima Microplate Reader (BMG Labtech). In both cases, cells were subjected to OGD with the OGD medium plus 5 *μ*M 2,7-dichlorofluorescin diacetate (DCF-DA; Sigma). The OGD medium was replaced by culture medium plus 5 *μ*M DCF-DA, cells were incubated in standard culture conditions, and experimental analysis was completed within 1 h.

### 2.6. NO Assay

HUVEC, SH-SY5Y, BV2, and HC-VSMC cells were seeded on T-25 flasks (approximately 5 × 10^6^ cells/flask) and subjected to OGD. Control media, 12 h post-ischemic media and 24 h postischemic media were collected, and NO was measured using a nitrate/nitrite colorimetric assay kit (Cayman).

### 2.7. Immunofluorescence

Hippocampal cells (4 × 10^4^ cells/well) were seeded on 1.5% gelatin coated, 12 mm coverslips. Cells were challenged for 1 h with OGD and maintained in standard culture conditions up to 24 h. Cells were fixed and incubated for 2 h at room temperature (RT) with 1 : 500 antinitrotyrosine polyclonal Ab (Chemikon), followed by incubation with 1 : 500 Alexa Fluor 488 goat anti-rabbit polyclonal Ab (Dako) for 1 h at RT. Nuclei were stained in blue with To-pro 3. The human cortex sample was cut at 3 *μ*m, deparaffinized at 70°C for 1 h, and washed with decreasing concentrations of ethanol. Antigen retrieval was performed with proteinase K at 40 *μ*g/mL in a 1 : 1 glycerol and TE buffer solution. Immunostaining was performed with 1 : 200 mouse monoclonal anti-NTyr Ab (Cayman Chem) for 2 h at RT, followed by 1 : 1000 Alexa555-bound anti-mouse as secondary Ab (Dako) overnight at 4°C. Sections were stained with To-pro 3 to identify the nuclei. All the coverslips were mounted with Mowiol. Images were taken with a Leica TCS SP confocal microscope and analyzed with Leica confocal software (Leica). 

### 2.8. Protein Identification by Western Blot

Cell cultures were lysed on ice with a solution containing 1 M Tris-HCl, 1% Nonidet P-40, 150 mM NaCl, 5 mM EDTA, 1 mM sodium orthovanadate, 1 mM dithiothreitol, pH 7.4, and a protease inhibitor cocktail (Roche). Protein concentration was determined by Bio-Rad protein assay. Protein samples were analyzed using 3–8% Tris-acetate gels (Invitrogen) for NOS detection. Gels were run at 150 V for 1 h and transferred to nitrocellulose membranes (Millipore) at 100 V for 2 h. Membranes were blocked in Tween 20-Tris buffer solution (100 mM Tris-HCl, 150 mM NaCl, and pH 7.5; 0.1% Tween, 5% milk) and incubated for 2 h at 25°C with 1 : 1,000 anti-nNOS (Santa Cruz Biotechnology), anti-iNOS (Santa Cruz Biotech.), and anti-eNOS (Santa Cruz Biotech.) Abs. Peroxidase-conjugated donkey anti-rabbit and anti-mouse Abs (Amersham Bioscience) were used as secondary Abs at 1 : 5,000 for 1 h at 25°C. Bands were visualized with Super Signal (Pierce) and Amersham Bioscience Hyperfilm ECL kit.

### 2.9. RNA Isolation and Reverse Transcriptase-Polymerase Chain Reaction (RT-PCR) Analysis

Total RNA from cell cultures was isolated using Trizol reagent (Invitrogen), following the manufacturer instructions. Briefly, 1 *μ*g of RNA was applied to RT-PCR using the OneStep RT-PCR Kit (Qiagen, Hamburg, Germany). The *iNOS*-specific primers for mice were 5′-CCATCACTGTGTTCCCCC-3′ and 5′-AAGGTGGCAGCATCCCC-3′ (Genbank accession number: NM_010927). The *iNOS*-specific primers for humans were 5′-CTGCTTGAGGTGGGCGG-3′ and 5′-GTGACTCTGACTCGGGACGCC-3′ (NM_000625). The *eNOS*-specific primers were 5′-CAAGTATGCCACCAACCGGG-3′ and 5′-ACTGAAGGGGGCTGCGG-3′ (NM_000603). The *nNOS*-specific primers were 5′-GAGAAGGAGCAGGGGGGG-3′ and 5′-CACATTGGCTGGGTCCCC-3′ (NM_000620). These primers were used to amplify the different *NOS. *Hypoxanthine phosphoribosyltransferase- (*hPRT-*) specific primers (5′-GGCCAGACTTTGTTGGATTTG-3′ and 5′-TGCGCTCATCTTAGGCTTTGT-3′; NM000194) were used as positive control. Negative control was performed in the absence of oligonucleotide primers. Results were analyzed with Image Gauge software (Fuji Photo Film Co.).

### 2.10. Cell Viability Assay

Cells were seeded in 96-well plates at a density of 8,000 cells/100 *μ*L per well and treated with 3-morpholinosydnonimine hydrochloride (SIN-1), sodium nitroprussiate (SNP), and/or H_2_O_2_, as described in the corresponding figures. Cells were incubated for 6 or 24 h at 37°C, and cell viability was measured by the 3-(4,5-dimethylthiazol-2-yl)-2,5-diphenyltetrazolium bromide (MTT) reduction method. Absorbances at 540 and 650 nm were determined in a Microplate Reader (Bio-Rad) and expressed as percentage of control.

### 2.11. Statistical Analysis

Data were expressed as the mean ± SEM of the values from the number of experiments indicated in the corresponding figures. Data were analyzed using one-way ANOVA with Bonferroni *post hoc* analysis.

## 3. Results

### 3.1. Ischemia Induces an Increase in Nitrotyrosination, Reactive Oxygen Species (ROS), and NO Production in Brain Cells

**Figure 1:**
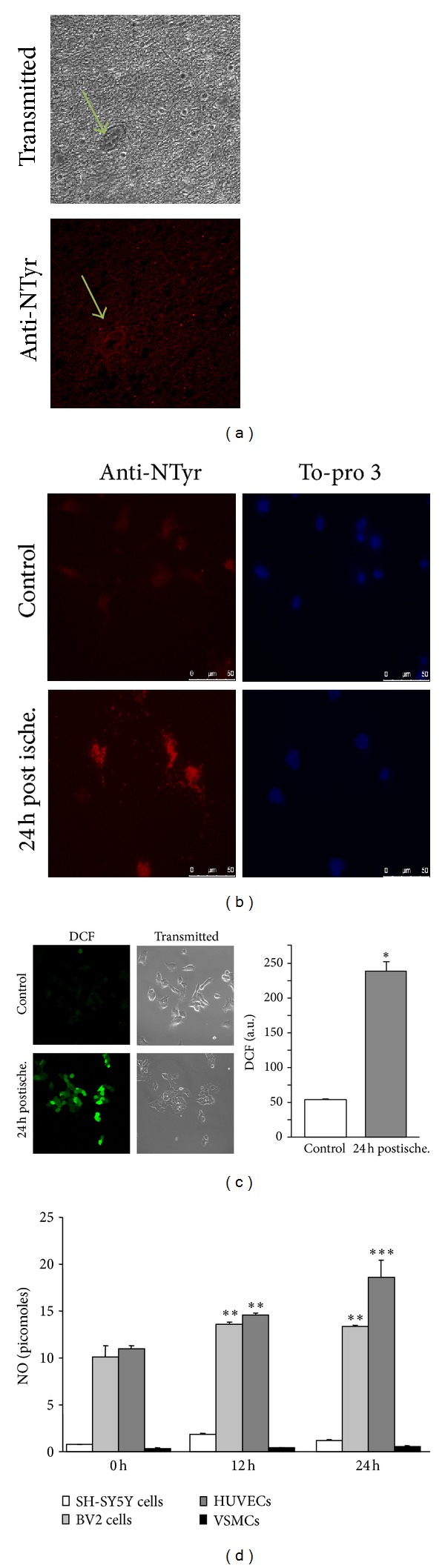
*In vitro* ischemia induces nitro-oxidative stress. (a) A representative image of nitrotyrosination (bottom image, stained in red) and bright field (top image) in a section of cortex from a stroke brain analyzed by immunohistochemistry. The arrows indicate blood vessels. (b) Primary hippocampal cells were subjected to *in vitro* ischemia followed by reperfusion with normal medium for 24 h. Nitrotyrosine (red staining) was demonstrated by immunostaining. Nuclei are stained in blue with To-pro 3. (c) Human neuroblastoma cells were subjected to *in vitro* ischemia reperfused with normal medium for 24 h. Free radical production was detected by DCF fluorescence and quantified. The mean fluorescence of DCF represents the levels of ROS. Data are mean ± SEM values of 4 independent experiments. *P < 0.05 versus control. (d) NO production (expressed in picomoles) was measured in neurons, microglia, endothelial, and vascular smooth muscle cells challenged with *in vitro* ischemia and later reoxygenated with normal growing medium containing glucose at 0, 12, and 24 h. Data are mean ± SEM values of 4 independent experiments. **P < 0.01; ***P < 0.001 versus controls at 0 h.

A strong labeling for nitrotyrosination was observed in the cortex from a stroke patient, not only in the brain parenchyma but also in the tunica intima and media of the blood vessel (Figure 1(a)). Neurons are very sensitive to oxidative stress. Therefore, we analyzed the protein nitrotyrosination after ischemia in mature mouse hippocampal neurons (Figure 1(b)). We assayed the effect of ischemia by subjecting the hippocampal neurons to OGD for 1 h, followed by reoxygenation. Neurons were maintained in a growth medium containing glucose for 24 h. We observed that hippocampal neurons subjected to ischemia had high levels of nitrotyrosination. The presence of nitrotyrosinated proteins demonstrated the existence of nitro-oxidative stress resulting from ischemia. 

To study free-radical production after ischemia, we exposed human neuroblastoma cells (SH-SY5Y) to OGD. After 1 h, SH-SY5Y cells showed a burst in free radical production, measured by DCF oxidation (Figure 1(c), right) and visualized by confocal microscopy (Figure 1(c), left).

In addition to neurons, other brain cell types are found in the ischemic area; all cell types could contribute to the damage by producing NO. After the ischemic challenge, microglia and endothelial cells showed an increase in NO production (Figure 1(d)) that persisted up to 24 h. Endothelial cells and microglia were the highest NO producers in both baseline conditions and after ischemia; SH-SY5Y and brain vascular myocytes had no effect on NO release due to ischemia (Figure 1(d)). 

### 3.2. Protein and mRNA Levels of NOS Types Are Modified in Brain Cells after Ischemia

**Figure 2:**
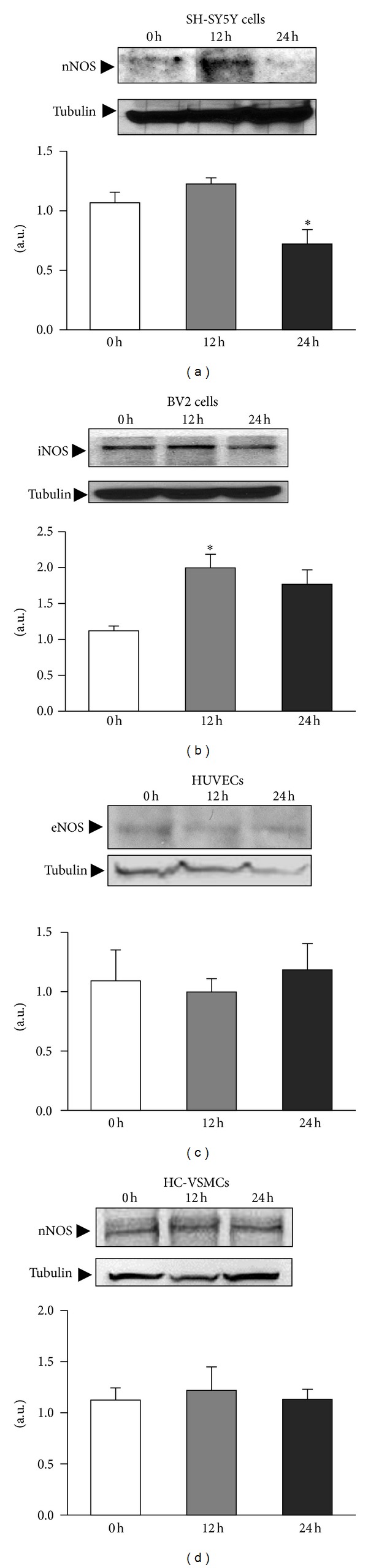
NO is produced in different cell types by the different NOS types. Cells were challenged with ischemia and iNOS, eNOS, and nNOS protein levels were studied immediately at time 0, 12, and 24 h after the ischemic challenge. Densitometric analysis of the bands quantified NOS expression relative to tubulin in all cell types. Data are mean ± SEM values of 6 experiments for microglia and 3 experiments for neurons, endothelial cells, and myocytes. *P < 0.05 versus controls at 0 h.

**Figure 3:**
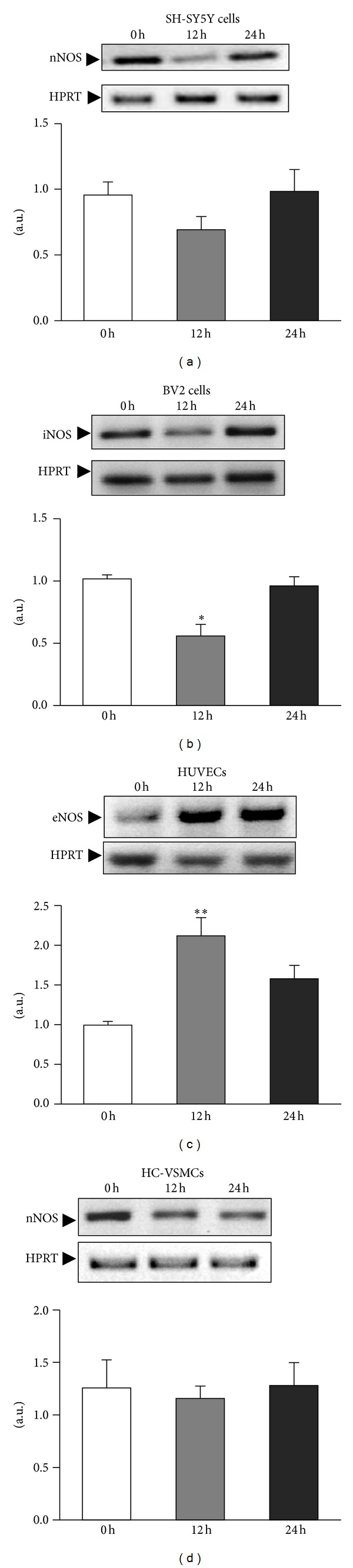
Differential expression of mRNA NOS types occurs in different cell types. Cells were challenged with ischemia, and *iNOS*, *eNOS,* and *nNOS* mRNA expression were studied immediately at time 0, 12 and 24 h after the ischemic challenge. The expression of mRNA was assessed by semiquantitative RT-PCR, and bands were quantified by *HPRT* densitometric analysis in all cell types. Data are mean ± SEM values of 3 experiments in all cell types. *P < 0.05; **P < 0.01 versus controls at 0 h.

NO production is dependent on the expression and activity of the different NOS. For this reason we examined the effect of ischemic insult on the protein and mRNA levels of *nNOS*, *eNOS,* and *iNOS*. In SH-SY5Y cells, we studied nNOS (Figure 2(a)). At 24 h after ischemia, we observed a significant reduction in nNOS protein (*P* < 0.05), but *nNOS* mRNA levels were not significantly affected (Figure 3(a)). 

 The main responsibility for the NO burst occurring after brain ischemia has classically been attributed to microglial cells. We studied the behavior of iNOS protein and mRNA in microglial cells (BV2) challenged with *in vitro* ischemia (Figure 2(b)). We found that iNOS is present in non-stimulated control microglial cells. Moreover, iNOS expression increased significantly at 12 h after the ischemic challenge (*P* < 0.005). The analysis of *iNOS* mRNA revealed, once again, a drop in the *iNOS* mRNA levels at 12 h (*P* < 0.05) that was normalized 24 h after the ischemic insult (Figure 3(b)).

 Regarding the expression of eNOS in endothelial cells (HUVEC), we did not see any significant change in the protein content after *in vitro* ischemia (Figure 2(c)), whereas the mRNA levels are upregulated after the ischemic challenge (*P* < 0.05; Figure 3(c)).

 The iNOS and nNOS expression has been reported in systemic rat vascular smooth muscle cells [25–29]. Neither iNOS protein nor *iNOS* mRNA was detected in myocytes (primary cultures of HC-VSMC) in basal conditions or after ischemia (data not shown); nNOS protein and mRNA were clearly identified (Figure 2(d)). nNOS protein is constitutively present in cerebral vascular myocytes. We did not observe any difference in *nNOS* (Figure 2(d)) and mRNA levels (Figure 3(d)) following exposure to OGD medium.

### 3.3. ONOO^−^ Induces Cell Death

**Figure 4:**
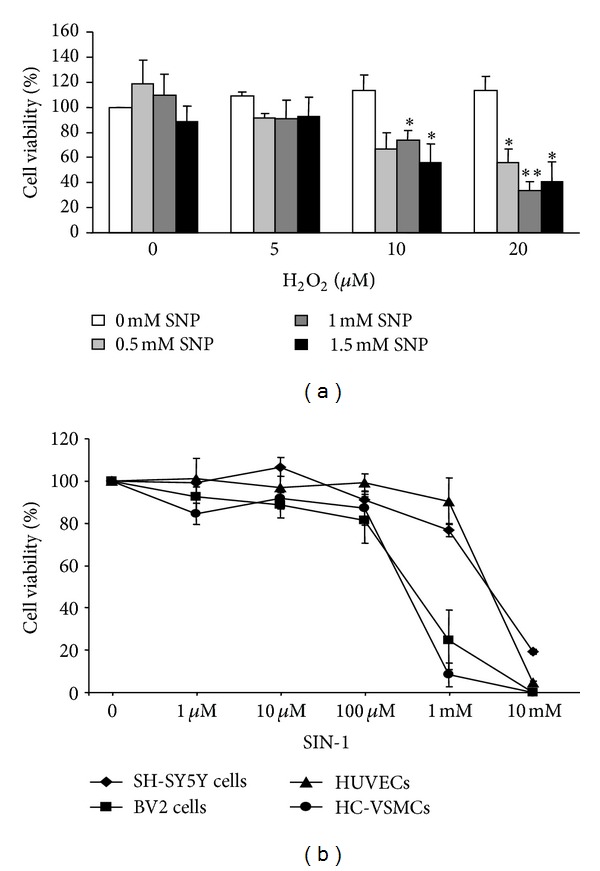
ONOO^−^ induces cell death. (a) Human neuroblastoma cells were treated with increasing concentrations of a NO donor (SNP) and H_2_O_2_ (free radical source). Cells were incubated for 6 h, and cell viability was assayed by MTT reduction. Data are expressed as percentage of control cells. Data are mean ± SEM values of 7 experiments. *P < 0.05; **P < 0.01 versus controls without SNP. (b) Cells were challenged with increasing concentrations of the ONOO^−^ donor SIN-1, and cell viability was assayed by MTT reduction.

We used a NO donor (SNP) to demonstrate that NO alone is not toxic to human neuroblastoma cells at high physiological concentrations (Figure 4(a)), tested up to 1.5 mM SNP (Figure 4(a)). However, when SNP was applied together with nontoxic concentrations of H_2_O_2_ (an oxidative stress source when metabolized by cells), we obtained neurotoxicity due to the formation of ONOO^−^ at 10 (*P* < 0.05) and 20 (*P* < 0.05 and *P* < 0.005) *μ*M H_2_O_2_, even with the lowest SNP concentration (0.5 mM), which is closer to the physiological concentrations obtained in NO burst production.

 The ONOO^−^ donor (SIN-1) was assayed at increasing concentrations in the different cell types (Figure 4(b)). The threshold to cause cytotoxicity was at 1 mM SIN-1 in endothelial cells and neuroblastoma cells. Myocytes and microglial cells were more susceptible to ONOO^−^, with the cytotoxicity threshold at 100 *μ*M SIN-1.

## 4. Discussion

Stroke generates a cascade of molecular events as a consequence of arrested blood supply [30]. Immediately after the first minutes of ischemic cerebral damage, the activity of eNOS becomes elevated in an attempt to improve blood supply [31]. Our data demonstrate that NO is released after *in vitro* ischemia in brain cells. The NO production is activated up to 24 h after ischemia, mainly in endothelial and microglial cells. At first it might constitute a homeostatic response to increase blood perfusion, but studies performed in iNOS and nNOS knockout (KO) mice suggest that the activation of these two NOS after ischemia is highly harmful to brain cells [32, 33].

The regulation of NO levels depends on the levels and expression of the different NOS. The activity of constitutive NOS (eNOS and nNOS) is regulated by intracellular calcium that increases in response to ischemic challenge. iNOS is regulated at the transcriptional level because it lacks the regulatory arm activated by Ca^2+^-calmodulin. Its transcription is activated by various stimuli, including LPS [34, 35].

We found significantly increased protein levels of iNOS in BV2 cells at 12 h after *in vitro *ischemia, and baseline levels were not totally recovered at 24 h. The regulation of mRNA expression for *iNOS* (BV2 cells) showed a decrease in mRNA levels at 12 h after ischemia, which correlates with high protein expression. The high production of NO by microglial cells after an ischemic challenge would be related to the nonspecific response of these cells to activation, as occurs with other immune cells [36] before phagocytosis of the cellular debris produced by postischemia necrosis [37]. This NO will not contribute to vasodilation but will mostly react with superoxide anion to form peroxynitrite. The iNOS KO mice had less neuronal death after ischemic stroke [32].

Postischemic protein levels (eNOS in HUVECs and nNOS in SH-SY5Y and HC-VSMCs) had not changed at 24 h. The *eNOS* mRNA was overexpressed at 12 h, which strongly suggests an inhibitory translational control of eNOS expression after ischemia. In one study, neuronal death increased in eNOS KO mice after a stroke [38], suggesting a protective role for this enzyme that is likely due to its direct control of blood flow. On the other hand, *nNOS* mRNA levels in SH-SY5Y and HC-VSMCs did not change during postischemic observation.

Oxidative stress plays a key role in ischemic-reperfusion situations [13] because mitochondrial dysfunction leads to a burst in free radical production that cannot be scavenged by the constitutive antioxidant cellular defense systems [39]. The large amount of free radicals produced during ischemia not only scavenges NO but also transforms it into the toxic ONOO^−^ [17], which yields to the protein nitrotyrosination and cell death observed in the present study. Although ONOO^−^ is a short lived molecule [40], its ability to diffuse through biological membranes can spread its harmful effects into neighboring cells and tissues. This critical process defines survival or death in the penumbra area. Consequently, we analyzed the effects of NO, H_2_O_2_, and NO plus H_2_O_2_ on neuronal survival. Neurotoxicity was only induced when cells were challenged with NO plus H_2_O_2_, which will produce peroxynitrite. Furthermore, we assayed increasing concentrations of a peroxynitrite donor, SIN-1, on all the brain cell types. We found a similar pattern of cell viability for all the cell types at low micromolar concentrations but a major cytotoxicity for glial cells and HC-VSMCs at 1 mM SIN-1. This effect may be related to a lower antioxidant defense. Further work is needed to investigate the possible protective role of antioxidants in the prevention of ischemic damage [41].

## 5. Conclusion

Our work demonstrates that brain ischemia induces nitro-oxidative stress that produces protein nitrotyrosination. The high production of peroxynitrite after ischemia will cause neuronal death and dramatically affect the survival of other brain cells.

## Nonstandard Abbreviations Used

Ab: Antibody
BSA: Bovine serum albumin
BV2: Mouse microglial cells
eNOS: Endothelial nitric oxide synthase
FBS: Fetal bovine serum
HC-VSMC: Human cerebral vascular smooth myocytes
hPRT: Hypoxanthine phosphoribosyltransferase
HUVEC: Human umbilical vein endothelial cells
iNOS: Inducible nitric oxide synthase
MTT: 3-(4,5-Dimethylthiazol-2-yl)2,5-diphenyltetrazolium bromide
NO: Nitric oxide
NOS: Nitric oxide synthase
O_2_^−^: Superoxide anion
OGD: Oxygen-glucose deprivation
ONOO^−^: Peroxynitrite anion
RT: Room temperature
RT-PCR: Reverse transcriptase-polymerase chain reaction
SH-SY5Y: Human neuroblastoma cells
SIN-1: 3-Morpholinosydnonimine hydrochloride.
